# Soluble amyloid triggers a myeloid differentiation factor 88 and interferon regulatory factor 7 dependent neuronal type-1 interferon response *in vitro*

**DOI:** 10.1186/s12974-015-0263-2

**Published:** 2015-04-12

**Authors:** Myles Robert Minter, Bevan Scott Main, Kate Maree Brody, Moses Zhang, Juliet Marie Taylor, Peter John Crack

**Affiliations:** Department of Pharmacology and Therapeutics, University of Melbourne, 8th floor, Medical building, Grattan St, Parkville, Melbourne, 3010 VIC Australia

**Keywords:** Type-1 interferon, Neuro-inflammation, Cytokines, JAK-STAT, Toll-like receptor, Myd88, IRF7, Amyloid, Alzheimer’s disease

## Abstract

**Background:**

Neuro-inflammation has long been implicated as a contributor to the progression of Alzheimer’s disease in both humans and animal models. Type-1 interferons (IFNs) are pleiotropic cytokines critical in mediating the innate immune pro-inflammatory response. The production of type-1 IFNs following pathogen detection is, in part, through the activation of the toll-like receptors (TLRs) and subsequent signalling through myeloid differentiation factor-88 (Myd88) and interferon regulatory factors (IRFs). We have previously identified that neuronal type-1 IFN signalling, through the type-1 interferon alpha receptor-1 (IFNAR1), is detrimental in models of AD. Using an *in vitro* approach, this study investigated the TLR network as a potential production pathway for neuronal type-1 IFNs in response to Aβ.

**Methods:**

Wildtype and Myd88^−/−^ primary cultured cortical and hippocampal neurons were treated with 2.5 μM Aβ1-42 for 24 to 72 h or 1 to 10 μM Aβ1-42 for 72 h. Human BE(2)M17 neuroblastoma cells stably expressing an IRF7 small hairpin RNA (shRNA) or negative control shRNA construct were subjected to 7.5 μM Aβ1-42/Aβ42-1 for 24 to 96 h, 2.5 to 15 μM Aβ1-42 for 96 h or 100 ng/ml LPS for 0.5 to 24 h. Q-PCR was used to analyse IFNα, IFNβ, IL-1β, IL-6 and TNFα mRNA transcript levels. Phosphorylation of STAT-3 was detected by Western blot analysis, and cell viability was assessed by MTS assay.

**Results:**

Reduced IFNα, IFNβ, IL-1β, IL-6 and TNFα expression was detected in Aβ1-42-treated Myd88^−/−^ neurons compared to wildtype cells. This correlated with reduced phosphorylation of STAT-3, a downstream type-1 IFN signalling mediator. Significantly, Myd88^−/−^ neuronal cultures were protected against Aβ1-42-induced neurotoxicity compared to wildtype as determined by MTS assay. Knockdown of IRF7 in M17 cells was sufficient in blocking IFNα, IFNβ and p-STAT-3 induction to both Aβ1-42 and the TLR4 agonist LPS. M17 IRF7 KD cells were also protected against Aβ1-42-induced cytotoxicity.

**Conclusions:**

This study confirms that the neuronal type-1 IFN response to soluble amyloid is mediated primarily through TLRs. This production is dependent upon Myd88 and IRF7 signalling. This study suggests that targeting this pathway to modulate neuronal type-1 IFN levels may be beneficial in controlling Aβ-induced neurotoxicity.

**Electronic supplementary material:**

The online version of this article (doi:10.1186/s12974-015-0263-2) contains supplementary material, which is available to authorized users.

## Introduction

Alzheimer’s disease (AD) is the most common cause of dementia with over 44 million current sufferers worldwide. Intracellular hyperphosphorylated tau [1] protein and intercellular amyloid-β (Aβ) plaque deposition [2] within the diseased brain remain the key histological abnormalities driving the proteinopathy, contributing to pyramidal neuronal loss. Features of neuro-inflammation are also evident in the AD brain. Activation and recruitment of microglia to Aβ plaque deposits [3], reactive astrogliosis [4] and elevated pro-inflammatory cytokine load [5] have all been identified in human postmortem tissue and in both *in vivo* and *in vitro* models of AD. Severe neuro-inflammation, involving elevated pro-inflammatory cytokine load, can induce cellular apoptosis directly but can also alter the dynamics of physiological cell processes including the classical amyloid precursor protein (APP) processing pathway [6]. Tumour necrosis factor alpha (TNFα) regulates *in vitro* Aβ production and processing by triggering alterations in beta-site APP cleavage enzyme-1 (BACE-1) activity [7]. In addition, direct TNFα-induced neurotoxicity mediated by interleukins (IL) also plays a major role in neurodegenerative disease [8]. Aβ remains a potent endogenous agonist for pro-inflammatory cytokine production leading to activation of cultured microglia [9]. Recently, it has been reported that the amyloid-dependent activation of the NALP3 inflammasome *in vivo* is a critical pathway in producing IL-1β and triggering a multi-faceted neuro-inflammatory response [10]. Our laboratory recently identified a role for type-1 interferon alpha receptor-1 (IFNAR1)-mediated signalling in Aβ-driven neuro-inflammation [11], known to interplay with NALP3 inflammasome activation [12,13]. The consequential cytokine storm triggers microglial activation and reactive astrogliosis creating a deleterious self-perpetuating neuro-inflammatory response by contributing to both excessive Aβ production and direct neuro-degeneration.

The toll-like receptor signalling (TLR), a subset of the pattern recognition receptor (PRR) super-family, remains a major source of pro-inflammatory cytokine production. There are 13 TLR subtypes currently identified in humans and mice combined. Apart from endosomal TLR3 and TRIF-dependent TLR4 activation, all signalling is dependent upon the myeloid differentiation factor-88 (Myd88) adaptor protein [14]. Subsequent TLR signalling leads to activation of interferon regulatory factors (IRFs) and NFκB that in turn drives pro-inflammatory cytokine production [15,16]. Whilst receptor density is greatest in the lung and spleen, these receptors are also widely expressed in the brain albeit at lower levels [17]. Resident microglia express all TLR subtype mRNA to facilitate their role as the brain’s ‘macrophage’. Depending upon various stimuli from the CNS microenvironment, these polarising cells can coordinate an M1 (neuro-protective) or M2 (reparative) innate immune response. It is widely accepted that microglia are critical for CNS inflammation; however, neurons themselves also express all TLR subtypes [18] and actively contribute to the pathogen-induced response. Principally, the TLR system is responsible for detecting foreign pathogen components (lipopolysaccharides and viral genomic material) as well as endogenous material released from damaged tissue (heat shock proteins, hyaluronic acid and mRNA). Detection of these ligands initiates the commencement of an innate immune response by which microglia, astrocytes and indeed neurons produce cytokines, triggering removal of the foreign or damaged material. Aβ has been identified as a novel endogenous ligand for many TLRs in the brain, triggering a neuro-inflammatory response (reviewed in [19]). Components of the TLR2 and TLR4 receptor complex are required for microglial detection of Aβ, mediating their phagocytic capacity [20,21]. Complete knockout of Myd88 in the APP_SWE_/PS1_ΔE9_ mouse model of AD ameliorated Aβ plaque burden, silenced microglial activation and reactive astrogliosis [22], and rescued some cognitive impairment [23]. In contrast, Myd88 heterozygous mice breed with APP_SWE_/PS1_ΔE9_ mice displayed significant spatial memory deficits in the T-water maze paradigm and displayed elevated soluble Aβ levels [24]. TLR4 mediates cytokine production in APP_SWE_/PS1_ΔE9_ mice [25], and mutations to this PRR confer reductions in microglial activation and amyloid phagocytosis [26]. The Aβ-induced and TLR-mediated inflammatory response is critical in amyloid processing and disease progression; however unlike glia, how neurons conduct this response remains unclear.

Type-1 interferons (IFNs), comprised of IFNα and IFNβ, are cytokine proteins that play an important role in host immune response to infections, pathogens and various diseases (reviewed in [27]). These pleiotropic cytokines are produced via activation of numerous PRRs including the TLR network, retinoic acid-inducible gene-1 (RIG-1), melanoma differentiation-associated protein-5 (MDA-5) and interferon gamma-inducible protein-16 (IFI-16) [28,29]. Type-1 IFNs can drive a pro-inflammatory response by activating the JAK-STAT pathway, leading to secretion of hallmark neuro-inflammatory cytokines TNFα, IL-6 and IL-1β. The myriad of cytokines and chemokines secreted provides a chemo-attractive environment, permitting cellular infiltration and inflammatory progression. Importantly, in the absence of type-1 IFN production and signalling, this inflammatory response fails to develop [30,31]. Although the Tyk-2/STAT pathway has been linked to soluble Aβ1-42 neurotoxicity [11,32], much remains unclear about type-1 IFN signalling in various cell types, including those in the brain.

It is critical to note that despite the complexity of type-1 IFN production, it all remains IRF dependent. The IRFs are well-characterised transcriptional regulators of the type-1 IFNs of which there have been nine mammalian isoforms identified. Of these nine, IRF3 and IRF7 are the key regulators of type-1 IFN production and thus play a central role in innate immunity [33]. Specifically, IRF7 activation is required for IFNα production resulting in an effective immune response to a wide variety of pathogens [33]. IRF7 is localised to the cell cytoplasm in an inactive state and undergoes phosphorylation, interacts with various co-activators and translocates to the nucleus upon pathogenic insult [34]. Recently, it has been reported that IRF7 and other type-1 IFN-regulated genes are commonly mutated amongst the human population, altering the efficacy of an innate immune response to various pathogens [35]. Furthermore, the type-1 IFN response in ageing mice reduces BDNF levels and doublecortin positive cells in the dentate gyrus, shown to negatively influence cognition and hippocampal neurogenesis [36]. The TLR network is primarily responsible for the detection of pathogens and this remains a major process responsible for IRF7 activation (reviewed in [37]). This activation is mediated by critical adaptor proteins TRIF, TRAF, IRAK and Myd88 [38-41] and permits a positive feedback loop by which type-1 IFN production drives further expression of IRF7 [42,43], exacerbating the innate immune response. The Aβ peptide is detected, but not solely, by the TLR network and has potential to activate IRF7 in this manner, but how this unrecognised signalling influences the neuro-inflammatory response to amyloid has yet been addressed.

Recently, our laboratory reported the involvement of type-1 IFNs in the neuro-inflammatory response in AD [11]. Specifically, we identified that Aβ1-42 initiates a deleterious type-1 IFN-mediated response in primary cultured neurons. This implicates the importance of neurons, not just microglia and astrocytes, in the inflammatory response to amyloid. In the current study, primary cultured murine neurons and human BE(2) M17 neuroblastoma cells were used to investigate the TLR network as a potential source of this type-1 IFN response. The neuronal type-1 IFN production in response to Aβ1-42 was mediated through Myd88 and IRF7. Furthermore, when TLR signalling was compromised by *in vitro* removal of Myd88 or IRF7, cultures displayed reduced type-1 IFN levels and protection against Aβ1-42-induced cell death. This study highlights the key role of TLR signalling in the neuronal neuro-inflammatory response to amyloid, critical when targeting this receptor system therapeutically.

## Materials and methods

### Antibodies

Primary antibodies used for Western blot analysis are the following: rabbit anti-p-STAT-3 (1:1,000, Cell Signalling, 9145S, Cell Signalling Technology, Danver, MA, USA), rabbit anti-STAT-3 (1:1,000, Cell Signalling, 4904S), mouse anti-β-actin (1:5,000, Sigma-Aldrich, A5441, Sigma-Aldrich, St. Louis, MO, USA). Secondary antibodies used for Western blot analysis are the following: horseradish peroxidase (HRP)-conjugated goat anti-rabbit (1:1,000, Dako, P0448, Agilent Technologies, Santa Clara, CA, USA) and goat anti-mouse (1:1,000, Dako, P0447).

### Mice

All animal experiments complied with the regulatory standards of, and were approved by, the University of Melbourne, Faculty of Medicine, Dentistry and Health Sciences Animal Ethics Committee (Ethics #1212477). Myd88^−/−^ mice on a pure C57Bl/6 background were generated by and kindly sourced from Prof. Shizuo Akira [44] and were mated for E14 to E16 pregnancy. Wildtype C57Bl/6 time-mated females where sourced from the Animal Resources Centre (ARC, Western Australia).

### Mixed cortical and hippocampal neuron isolation

Mixed cortical and hippocampal neurons were isolated from embryonic day 14 to 16 embryos as previously described [45]. Briefly, cortices were dissected from the foetal brains and the meningeal layers removed. Tissue was then mechanically processed and treated with trypsin (Sigma-Aldrich, T9201) and DNAse (Sigma-Aldrich, D5025) in Krebs solution (supplemented with 0.3% BSA w/v and 176 nM MgSO_4_) to produce a single-cell suspension. Cells were allowed to adhere for 2 h, to remove potential contaminating fibroblasts and glia, before cultures were plated at 10^6^ cells/ml in plating media (Neurobasal media containing B27 supplement (Gibco, 17504–044, Life Technologies, Carlsbad, CA, USA) and 2% FBS). The following day, FBS was removed from the media and cultures were supplemented with fresh culture media (Neurobasal media containing B27 supplement) every 2 days until treatment. Cultures were treated at 7 days *in vitro*. Purity of cultures was confirmed to be >95% neurons by NeuN and GFAP staining to identify neurons and glia, respectively.

### M17 neuroblastoma cells

Human BE(2) M17 neuroblastoma cells (ATCC® number: CRL-2267™) were cultured in T75 flasks with culture medium (OptiMEM (Gibco), 5% FBS, 0.5% penicillin-streptomycin (Gibco)) at 37°C/5% CO2 until 90% confluent. Cells were plated at 2 × 10^5^ cells/ml in 6-cm dishes for 24 h prior to treatment.

### Generation of M17 IRF7 knockdown cell line

IRF7 knockdown (IRF7 KD) and negative control (NC) knockdown M17 cells were generated using commercially available small hairpin RNA (shRNA) plasmid constructs (Origene, Rockville, MD, USA). Briefly, M17 cells were transfected using Fugene®HD (Promega, Madison, WI, USA) with HuSH shRNA plasmids containing an IRF7 specific shRNA cassette or non-effective 29-mer scrambled shRNA cassette. Clonal cell lines were generated using the selectable marker puromycin (0.5 μg/ml, Gibco, A11138-03). Successful knockdown of IRF7 (>70%) was confirmed by QPCR where IRF7 levels in IRF7 KD M17 cells were compared to their NC M17 cell counterparts (Additional file 1: Figure S1).

### Amyloid-beta preparation and treatment

Amyloid peptide stocks were generated according to protocol by [46]. The amyloid-β (Aβ) 1-42 (peptide corresponding to amino acids 1 to 42, GenecBio, A-42-T-1) peptide was initially reconstituted in 1,1,1,3,3,3-hexafluoro-2-propanol at 0.5 mg/mL, lyophilised, and stored at −80°C until required. The peptide was then dissolved in a 5 mM NaOH vehicle and protein concentration determined by absorbance spectrophotometry at 214 nm as previously described [47]. This enhances the Aβ peptide solubility and lengthens the time frame in which toxicity is induced [46,48-51]. These preparations lead to globular aggregate structures that resemble the oligomeric confirmation rather than elongated fibril structures [52-54]. Primary neuronal cultures were then treated with 0.5 to 10 μM Aβ1-42 or vehicle for up to 72 h in treatment medium (Neurobasal media containing anti-oxidant free B27 supplement (Gibco, 10889038)). M17 NC shRNA and M17 IRF7 KD cell cultures were treated with 2.5 to 15 μM Aβ1-42 for up to 96 h in fresh serum-reduced culture medium (2% FBS). The final NaOH concentration in the culture medium was <5 nM and shown to be non-toxic.

### Lipopolysaccharide preparation and treatment

Lipopolysaccharides from *Escherichia coli* 026:B6 (LPS, Sigma-Aldrich, L8274) were initially reconstituted in phosphate-buffered saline (PBS) to yield a concentration of 1 mg/ml, and aliquots were stored at −80°C until required. M17 human neuroblastoma cells were then treated with 100 ng/ml LPS for 0.5 to 24 h in fresh serum-reduced culture medium (2% FBS).

### Protein extraction

Following treatment, primary neuronal and M17 cell cultures were collected via cell scraping in ice-cold PBS. After centrifugation at 5,000 g, cell pellets were sonicated in lysis buffer (50 mM tris, 1% Triton x-100, 1% SDS, PhosphoSTOP® and protease inhibitors (Roche, Basel, Switzerland), pH 7.4) and protein concentrations determined by Bradford assay (Bio-Rad, Hercules, CA, USA).

### Western blot analysis

A 50 μg of protein was resolved on 10% acrylamide SDS PAGE gels and transferred to polyvinylidene fluoride (PVDF) membranes by semi-dry transfer. Membranes were blocked with 5% w/v skim milk powder in TBS-T for 1 h at room temperature (≈20°C) before overnight incubation with primary antibodies at 4°C. Membranes were washed in TBS-T before being incubated with HRP-conjugated secondary antibodies (diluted in 5% (w/v) BSA in TBS-T) for 120 min at room temperature (≈20°C). After additional washing in TBS-T, signals were detected using an ECL™ prime Western blotting detection kit (Amersham, RPN2232, GE Healthcare, Little Chalfont, UK) and visualised using the ChemiDoc™ MP system (Bio-Rad).

### RNA isolation and cDNA synthesis

Cells were lysed in TRIzol® (Life Technologies, 15596018) and RNA isolated per manufacturer’s instructions with concentrations determined by the Nanodrop 1000 spectrophotometer (Thermo Scientific, Waltham, MA, USA). A 1 μg quantity of RNA was reversed transcribed into cDNA using a high-capacity cDNA reverse transcription kit (Applied Biosystems, Life Technologies, USA, 4368814) according to manufacturer guidelines. cDNA was diluted 1:3 in DEPC-treated dH_2_O for use in quantitative PCR (Q-PCR).

### Quantitative PCR (Q-PCR)

All Q-PCR was performed in standard 384-well plates using the 7900HT fast real-time PCR system (Applied Biosystems, Life technologies, USA). Taqman probes (Applied Biosystems, Life technologies, USA) were used to analyse huIFNα (Hs00256882_sl), huIFNβ (Hs01077958_sl), UBC (Hs00824723_m1), muGAPDH (Mm99999915_m1), muIFNβ (Mm00439552_s1), muIL-1β (Mm01336189_m1), muIL-6 (Mm00446190_m1) and muTNFα (Mm00443258_m1) under the following cycle parameters: 50°C for 2 min, 94.5°C for 10 min, (97°C for 30 s, 59.7°C for 60 s) × 40 repeats. The SYBR green system was used to analyse muIFNα and muGADPH using the following primer sequences (Geneworks, Hindmarsh, SA, Australia): GADPH forward: 5′ ATCTTCTTGTGCAGTGCCAGC 3′, GADPH reverse: 5′ ACTCCACGACATACTCAGCACC 3′, muIFNα forward: 5′ GCAATCCTCCTAGACTCACTTCTGCA 3′, muIFNα reverse 1: 5′ TATAGTTCCTCACAGCCAGCAG 3′, muIFNα reverse 2: 5′ TATTTCTTCATAGCCAGCTG 3′, muTLR2 forward: 5′ TGCTTTCCTGCTGAAGATTT 3′, muTLR2 reverse: 5′ TGTACCGCAACAGCTTCAGG 3′, muTLR4 forward: 5′ ACCTGGCTGGTTTACACGTC 3′, muTLR4 reverse: 5′ TGTCCAGAGACATTGCAGAA 3′. SYBR green Q-PCR was then performed under the following temperature regulations: 95°C for 20 min, (95°C for 30 s, 60°C for 90 s) × 40 repeats, 95°C for 15 min, 60°C for 15 min, 95°C for 15 min. Fold change in mRNA levels were then calculated using the ΔΔct method (2^-ΔΔCt^) [55]. Cycle threshold (Ct) values were obtained from the linear gradient section of each fluorescent PCR amplification curve. The Ct values of each individual reaction triplicate for genes of interest were then normalised back to the GAPDH housekeeping gene, yielding ΔCt values. The calculated ΔCt of the Aβ treatment groups were then normalised against respective genotype-specific vehicle control samples (ΔΔCt). As all ΔΔCt values were calculated from the linear gradient amplification curve, conferring 100% enzymatic efficiency, and PCR is an exponential process, ΔΔCt values were then converted to fold change (fold change = 2^(−ΔΔCt)^). All Q-PCR data expressed throughout this publication has been calculated using this method.

### MTS assay

Cell viability was measured by the cellular ability to metabolise 3-(4,5-dimethylthiazol-2-yl)-5-(3-carboxymethoxyphenyl)-2-(4-sulfophenyl)-2H-tetrazolium (MTS) (Promega) in the presence of phenazine methosulfate to a formazan product, as described previously [56]. Viability of treated samples was expressed as a percentage of light absorbance at 490 nm of the vehicle control, with all experiments performed in triplicate. Staurosporine (1 μM) was used to induce cellular apoptosis and used as a positive control for apoptotic cell death in the neuronal cultures.

### Statistical analysis

For all Q-PCR, MTS assay and Western blot densitometry data, an unmatched two-way ANOVA was performed for each time point/concentration with cellular genotype as the fixed variable. A Bonferroni *post hoc* test was then used to determine statistical significance (*p* < 0.05) between genotype groups. Graphical data is displayed as mean ± SEM.

## Results

### The type-1 IFN response to Aβ1-42 is Myd88 dependent in primary cultured neurons

**Figure 1:**
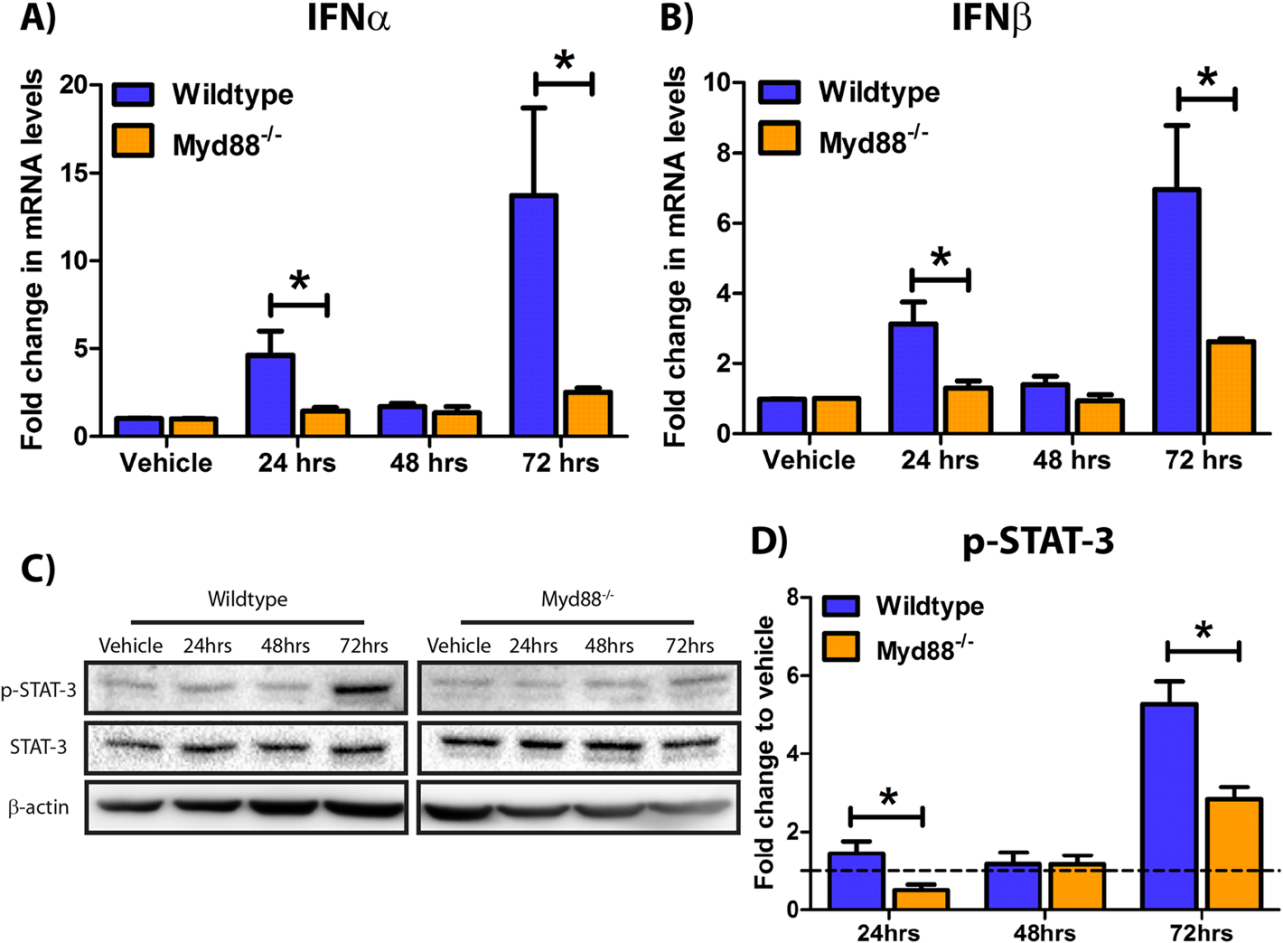
Myd88^−/−^ neurons display reduced type-1 interferon load and signalling in response to Aβ1-42 insult. Wildtype and Myd88^−/−^ primary cultured murine mixed cortical and hippocampal neuronal cultures were treated with 2.5 μM Aβ1-42 for 24 to 72 h. (**A**) IFNα and (**B**) IFNβ mRNA levels were analysed by Q-PCR (*p < 0.05, *n* = 5 to 6, unmatched two-way ANOVA, Bonferroni *post hoc* test). (**C**) STAT-3 tyrosine 705 phosphorylation of whole cell extracts was measured by Western blot. (**D**) Corresponding densitometry (*p < 0.05, *n* = 5, unmatched two-way ANOVA, Bonferroni *post hoc* test). For densitometry calculations, phosphorylation intensity was measured in arbitrary units (A.U.) and normalised to the STAT-3:β-actin intensity ratio. All graphical data is displayed as mean ± SEM with treatment time on the *x* axis. Full-size uncropped Western blots can be viewed in Additional file 4: Figure S2.

Previously, we have confirmed that neurons alone are capable of initiating a type-1 IFN response to soluble amyloid [11]. The TLR system has previously been implicated in the detection of soluble amyloid [57], and classical TLR signalling leads to production of type-1 IFNs. We confirm that TLR4 and TLR2, albeit at lower levels, are expressed in our primary cultured mixed cortical and hippocampal neurons (Additional file 2: Table S1). A critical adapter protein required for the majority of TLR-mediated signalling is Myd88; thus, we used Myd88^−/−^ neuronal cultures to investigate the source of type-1 IFN production in response to Aβ1-42. Wildtype and Myd88^−/−^ neurons were treated with 2.5 μM Aβ1-42 for 24 to 72 h. This Aβ1-42 concentration routinely gives ≈ 50% cell death, thus simulating amyloid neurotoxicity and allowing surviving cells to react to the neuro-inflammatory environment [11]. Q-PCR identified attenuated IFNα (4.6-fold vs. 1.5-fold at 24 h, 13.7-fold vs. 2.5-fold at 72 h, Figure 1A) and IFNβ (3.1-fold vs. 1.3-fold at 24 h, 7.0-fold vs. 2.6-fold at 72 h, Figure 1B) mRNA transcript levels in Aβ1-42-treated Myd88^−/−^ cultures compared to wildtype. Type-1 IFNs directly stimulate the JAK-STAT cascade to initiate pro-inflammatory cytokine transcription; hence, we investigated the phosphorylation of STAT-3 in these cultures. Western blot identified elevated tyrosine 705 phosphorylation of STAT-3, at 24 and 72 h, in Aβ1-42-treated wildtype neuronal cultures. Densitometry confirmed that this phosphorylation was significantly reduced in Myd88^−/−^ neuronal cultures (1.4-fold vs. 0.5-fold at 24 h, 5.3-fold vs. 2.8-fold at 72 h, Figure 1C,D, Additional file 3: Figure S3). Synthetic peptides [58] and potential endotoxin contaminants [59] possess the ability to act as DAMPs and in turn trigger an immune response. To confirm that the observed type-1 response is Aβ1-42 specific and not an artefact response from a foreign peptide, neuronal cultures were also treated with the control Aβ42-1 peptide. Significantly, neuronal Aβ42-1 treatment failed to generate type-1 IFN expression (Additional file 4: Figure S2A) and STAT-3 phosphorylation (Additional file 4: Figure S2B) in WT neurons indicating that this innate immune response is Aβ1-42 specific. This data confirms that neurons alone have the capacity to initiate a type-1 IFN response specifically against the Aβ1-42 peptide. This response is dependent upon Myd88 signalling, a critical adaptor protein in the TLR network.

### Hallmark pro-inflammatory type-1 IFN-regulated cytokine expression is reduced in Myd88^−/−^ neuronal cultures following Aβ1-42 insult

**Figure 2:**
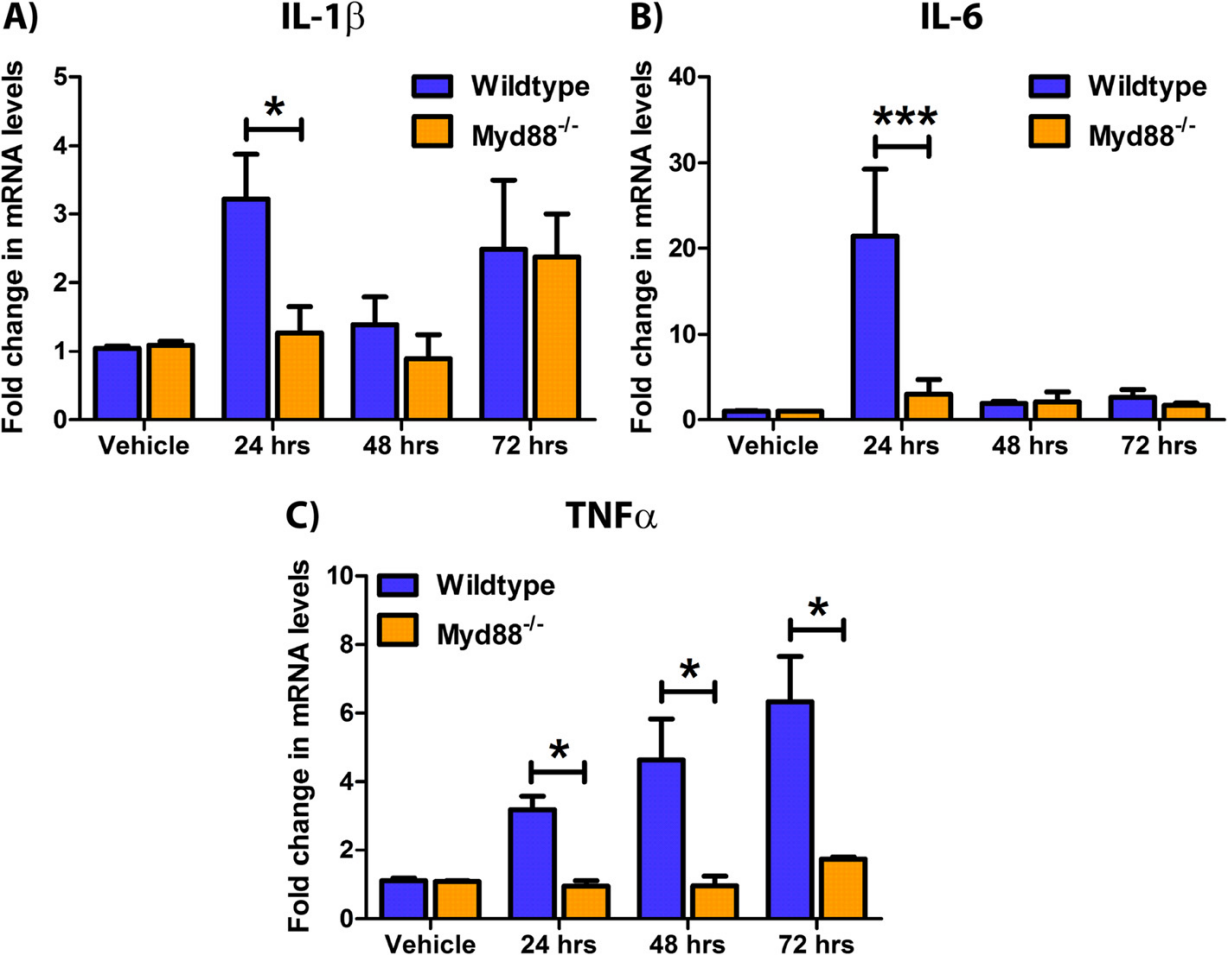
Removal of Myd88 in Aβ1-42-treated neuronal cultures confers reductions in hallmark pro-inflammatory cytokine secretion. Wildtype and Myd88^−/−^ primary cultured murine mixed cortical and hippocampal neuronal cultures were treated with 2.5 μM Aβ1-42 for 24 to 72 h. Q-PCR was performed to analyse (**A**) IL-1β, (**B**) IL-6, and (**C**) TNFα mRNA transcript levels (*p < 0.05, ***p < 0.001, *n* = 5 to 6, unmatched two-way ANOVA, Bonferroni *post hoc* test). All data is displayed as mean ± SEM with treatment time on the *x* axis.

The products of type-1 IFN-driven JAK-STAT signalling are a myriad of pro-inflammatory cytokines that in turn drive the innate immune response. Interleukins and TNFα are critical cytokines in potentiating this immune response and are upregulated in settings of amyloid pathology [60]; thus, we assessed their expression in Myd88^−/−^ neuronal cultures. Wildtype and Myd88^−/−^ neurons were treated with 2.5 μM Aβ1-42 for 24 to 72 h. Expression of the hallmark pro-inflammatory cytokines IL-1β (3.2-fold vs. 1.3-fold at 24 h, Figure 2A), IL-6 (21.4-fold vs. 3.0-fold at 24 h, Figure 2B) and TNFα (3.2-fold vs. 1.0-fold at 24 h, 4.6-fold vs. 1.0-fold at 48 h, 6.3-fold vs. 1.7-fold at 72 h, Figure 2C) were decreased in Myd88^−/−^ neurons compared to wildtype cultures in response to Aβ1-42. This data confirms a neuronal source of hallmark pro-inflammatory cytokine secretion in response to amyloid. This response, comprised of interleukins and TNFα, is governed by TLR-Myd88-dependent signalling.

### Removal of Myd88 confers protection against Aβ1-42 toxicity in primary cultured neurons

**Figure 3:**
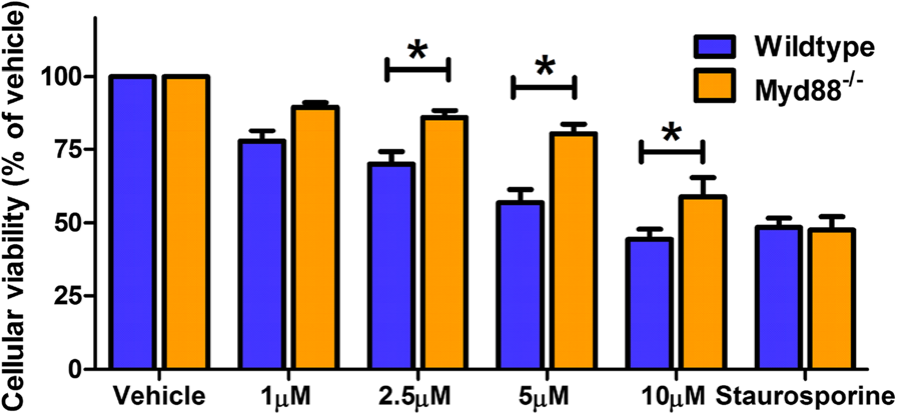
Myd88^−/−^ neurons are protected against soluble Aβ1-42 toxicity. Wildtype and Myd88^−/−^ neuronal cultures were treated with 1 to 10 μM Aβ1-42 for 72 h and an MTS assay for cell viability was performed (*p < 0.05, *n* = 9, unmatched two-way ANOVA, Bonferroni *post hoc* test). Staurosporine (1 μM) was used as a positive control for neuronal cell death. Data is displayed as mean ± SEM with treatment concentration on the *x* axis.

To investigate the overall effect of reducing type-1 IFN production and signalling through removal of Myd88, cultures were treated with 1 to 10 μM Aβ1-42 for 72 h and neuronal viability was measured by MTS assay. Myd88^−/−^ neurons were significantly less susceptible to Aβ1-42-induced neurotoxicity compared to wildtype cells (69.9% vs. 86.1% viability with 2.5 μM, 56.8% vs. 80.4% viability with 5 μM, 44.3% vs. 58.8% viability with 10 μM, Figure 3). No difference in viability between genotypes in response to the apoptosis-inducing staurosporine was identified. This finding suggests that a reduction in type-1 IFN and hallmark pro-inflammatory cytokines, via removal of Myd88, confers protection against soluble amyloid cytotoxicity.

### The type-1 IFN response to Aβ1-42 is IRF7 dependent and mimics an LPS-induced type-1 IFN signature in human BE(2) M17 neuroblastoma cells

**Figure 4:**
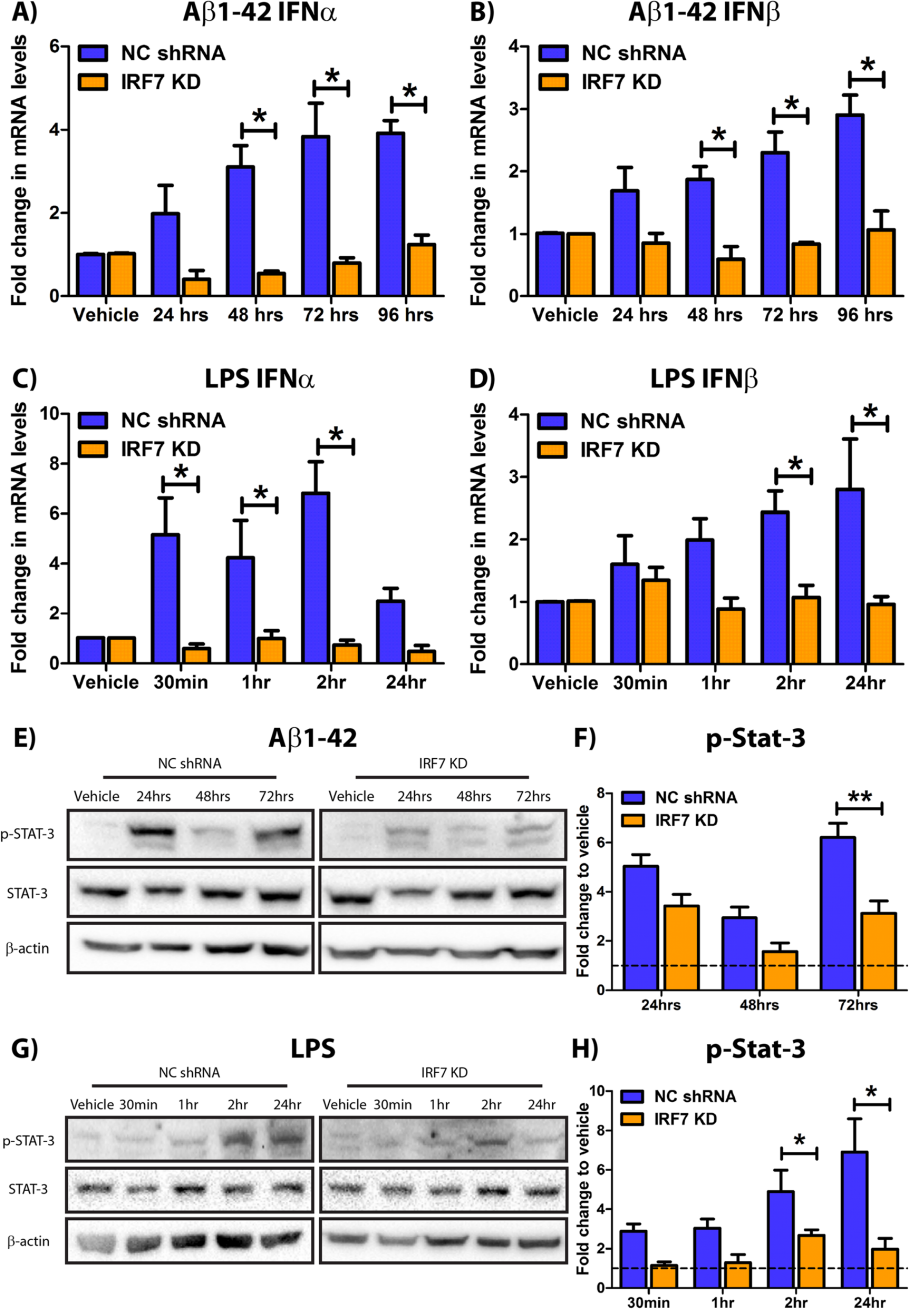
The neuronal type-1 IFN response to Aβ1-42 is IRF7 dependent and mimics an LPS-induced interferon signature. Human BE(2) M17 neuroblastoma cells, transfected with an IRF7 knockdown (KD) construct or negative control (NC) plasmid, were treated with 7.5 μM Aβ1-42 for 24 to 96 h. Q-PCR was performed to analyse (**A**) IFNα and (**B**) IFNβ mRNA levels post-treatment (*p < 0.05, *n* = 9 to 12, unmatched two-way ANOVA, Bonferroni *post hoc* test, alternative clone data can be viewed in Additional file 4: Figure S2). Cells were treated with 100 ng/ml LPS for 0.5 to 24 h and (**C**) IFNα and (**D**) IFNβ mRNA levels were analysed by Q-PCR (*p < 0.05, *n* = 7 to 9, unmatched two-way ANOVA, Bonferroni *post hoc* test). (**E**) The phosphorylation of STAT-3 in the Aβ1-42-treated cultures was analysed by Western blot analysis. (**F**) Corresponding densitometry. (**p < 0.01, *n* = 6, unmatched two-way ANOVA, Bonferroni *post hoc* test). (**G**) The protein lysates of the LPS-treated cells were also analysed for STAT-3 phosphorylation by Western blot with (**H**) corresponding densitometry (*p < 0.05, *n* = 12, unmatched two-way ANOVA, Bonferroni *post hoc* test). For densitometry calculations, phosphorylation intensity was measured in arbitrary units (A.U.) and normalised to the STAT-3:β-actin intensity ratio. All graphical data is displayed as mean ± SEM with treatment time on the *x* axis. Full-size uncropped Western blots of Aβ- and LPS-treated cultures can be viewed in Additional file 6: Figure S5 and Additional file 7: Figure S6, respectively.

IRF7 has been reported as the critical mediator of type-1 IFN production and is a major downstream signalling molecule in Myd88-dependent and independent TLR cascades [61]. To investigate if IRF7 is critical in soluble amyloid-driven type-1 IFN production in human M17 neuroblastoma cells, we established a stable IRF7 shRNA cell line and treated with 7.5 μM Aβ1-42 for 24 to 72 h. Q-PCR identified decreased IFNα (3.1-fold vs. 0.5-fold at 48 h, 3.8-fold vs. 0.8-fold at 72 h, 3.9-fold vs. 1.2-fold at 96 h, Figure 4A) and IFNβ (1.9-fold vs. 0.6-fold at 48 h, 2.3-fold vs. 0.8-fold at 72 h, 2.9-fold vs. 1.1-fold at 96 h, Figure 4B) mRNA transcript levels in Aβ1-42-treated M17 IRF7 KD cells compared to M17 NC shRNA cultures. This data is supported by similar findings using an alternative M17 IRF7 KD clonal cell line with 77% knockdown efficiency (Additional file 5: Figure S4). As the amyloid peptide is detected by many TLR isoforms, we utilised LPS to ascertain a TLR4-specific type-1 IFN response profile and compared this to the Aβ1-42 data. IRF7 KD shRNA and NC shRNA M17 cells were treated with 100 ng/ml LPS for 30 min to 24 h. Q-PCR showed decreased IFNα (5.2-fold vs. 0.6-fold at 30 min, 4.2-fold vs. 1.0-fold at 1 h, 6.8-fold vs. 0.7-fold at 2 h, Figure 4C) and IFNβ (2.4-fold vs. 1.1-fold at 2 hours, 2.8-fold vs. 1.0-fold at 24 hours, Figure 4D) mRNA levels in LPS-stimulated M17 IRF7 KD cells compared to NC shRNA cultures. Western blot analysis identified a trend for decreased STAT-3 phosphorylation levels at 24 h (5.0-fold vs. 3.4-fold, *t* = 2.064, DF = 5) and a significant reduction at 72 h (6.2-fold vs. 3.1-fold) post-Aβ1-42 treatment in M17 IRF7 KD cells compared to NC cultures (Figure 4E,F, Additional file 6: Figure S5). Similarly, LPS-induced STAT-3 phosphorylation levels were reduced at 2 h (4.9-fold vs. 2.7-fold) and 24 hours (6.9-fold vs. 2.0-fold) in the M17 IRF7 KD cells (Figure 4G/H, Additional file 7: Figure S6). This data confirms that both type-1 IFN responses to the peptide TLR ligand, Aβ1-42 and the TLR4 selective agonist, LPS, are IRF7 dependent in human BE(2) M17 neuroblastoma cells. This finding implies that TLR4 detection of amyloid and IRF7 signalling contributes to the net neuronal type-1 IFN response to soluble amyloid.

### Reduction of IRF7 levels confers protection against Aβ1-42 toxicity in human BE(2) M17 neuroblastoma cells

**Figure 5:**
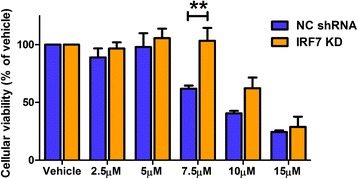
Knockdown of IRF7 in human BE(2) M17 neuroblastoma cells confers protection against Aβ1-42. Human BE(2) M17 neuroblastoma cells, transfected with an IRF7 knockdown (KD) construct or negative control (NC) plasmid, were treated with 2.5 to 15 μM Aβ-42 for 72 h and an MTS assay to measure cell viability was performed (**p < 0.01, *n* = 5, unmatched two-way ANOVA, Bonferroni *post hoc* test). All data is displayed as mean ± SEM with treatment concentration on the *x* axis.

Previously, and in the current study, we have reported that reducing type-1 IFN secretion in response to amyloid in neuronal cultures affords cellular protection [11]. Thus, we assessed the effect on cytotoxicity of decreased type-1 IFN production and signalling in response to Aβ1-42 using M17 IRF7 KD cells. M17 IRF7 KD cells and NC shRNA cultures were treated with 2.5 to 15 μM Aβ1-42 for 96 h, and an MTS assay was performed to evaluate cellular viability. M17 IRF7 KD cells were significantly protected against 7.5 μM Aβ1-42 (61.8% vs. 103.1% viability) compared to their NC shRNA counterparts with a protective effect also suggestive at 10 μM Aβ1-42 (40.5% vs. 62.3% viability, *t* = 2.128, DF = 4) (Figure 5). This data suggests that reducing IRF7-dependent production of type-1 IFNs in response to Aβ1-42 confers a protective phenotype in human BE(2) M17 neuroblastoma cells.

## Discussion

Deposition of the Aβ peptide and formation of insoluble plaques are hallmark pathologies associated with AD. However, the extent of plaque formation fails to correlate with disease progression [62] and therefore it has been considered that the soluble amyloid oligomers are the primary neurotoxic species contributing to the neuro-degeneration [63,64]. The precise mechanisms of soluble Aβ-induced neurotoxicity however are still poorly understood. Neuro-inflammation has been implicated in AD progression, but the exact contribution of each CNS-residing cell type in this response remains unclear. Microglia, the resident inflammatory cells of the CNS, remain critically important for the clearance of Aβ; however, it is ultimately the neuronal populations that respond detrimentally to the neuro-inflammatory environment in AD and are indeed the cells lost in the pathology [65,66]. We have previously established a role for type-1 IFNs in animal and cell models of Aβ toxicity [11]. Specifically, we identified neuronal populations as a source of type-1 IFN secretion in response to soluble Aβ insult. This current study investigated TLR signalling pathways as a potential origin for this neuronal type-1 IFN response to Aβ. Myd88 remains a critical adaptor protein in the majority of TLR pathways and drives IRF7 activation, in turn upregulating the expression and subsequent production of type-1 IFNs. Type-1 IFN secretion and downstream signalling via p-STAT-3 was downregulated in Myd88^−/−^ primary neurons following Aβ1-42 challenge. This finding correlated with reductions in hallmark pro-inflammatory cytokines IL-1β, IL-6 and TNFα and resulted in neuro-protection against soluble Aβ. We show that knockdown of IRF7 is sufficient to abolish the type-1 IFN response to both the selective TLR4 agonist LPS and the indiscriminate TLR ligand Aβ1-42 in human neuroblastoma M17 BE(2) cells. Furthermore, IRF7 knockdown resulted in protection against Aβ1-42 toxicity.

TLRs, a super-family of pattern recognition receptors, are the host’s primary detectors of foreign pathogens and mediate the initial type-1 IFN response in the innate immune response [67]. A myriad of TLR subtypes are capable of detecting Aβ and initiating an innate immune response against the neurotoxic peptide [57,68,69]. Myd88 is a critical adaptor protein for all TLR signalling except for TLR3 and Myd88-independent TLR4 activation. The involvement of Myd88 in animal models of amyloidosis remains controversial and somewhat conflicted. Mice over-expressing APP and PS1 on a Myd88^−/−^ background showed reduced soluble and insoluble amyloid load at 10 months correlating with reductions in reactive astrogliosis and microglial activation [22]. These same mice appeared to show no spatial learning improvement in the Morris water maze compared to APP_swe_/PS1_ΔE9_ mice alone but interestingly, removal of Myd88 was sufficient to improve this outcome regardless of transgene expression [23]. APP_swe_/PS1_ΔE9_ mice on a heterozygous Myd88 background also showed reduced plaque deposition but elevated soluble Aβ levels. These mice were found to have reduced brain IL-1β mRNA and had accelerated spatial memory deficits in the T-water maze [24]. Most recently, APP_swe_/PS1_ΔE9_ mice on a Myd88 deficient background showed no changes in amyloid levels or immune cell infiltration but displayed a reduced life expectancy [70]. All these reports ultimately focus on the astrocyte and microglial response to amyloid load and how Myd88 absence affects this process. Still, there remain conflicting conclusions arising from these animal models.

Neuro-inflammation is an intricate process with multiple signalling cascades and critical cellular processes driving pathogen clearance. Our study aimed to simplify the previously conducted *in vivo* studies and used a controlled *in vitro* approach to assess the impact of Myd88 signalling in response to soluble amyloid. In this study, we focus on the impact of Myd88 removal in neurons and how this may alter the type-1 IFN response to the amyloid peptide. Removal of Myd88 in primary neuronal cultures conferred reductions in type-1 IFNs and hallmark pro-inflammatory cytokines IL-1β, IL-6 and TNFα compared to wildtype when challenged with Aβ1-42. This reduction in pro-inflammatory cytokine expression in the Myd88^−/−^ cells also coincided with decreased p-STAT-3 levels, a downstream type-1IFN signalling mediator. This data supports the hypothesis that the TLR network is responsible for detecting soluble amyloid [19]. Furthermore, we hypothesise that the neuronal type-1 IFN response to amyloid occurs via a TLR-mediated mechanism. This type-1 IFN response is biphasic in nature which remains a common characteristic of the type-1 IFN signature displayed upon pathogenic infection [71-73]. This release begins from 24 h post-Aβ insult, returns to baseline by 48 h and enters a secondary ramping phase from 72 h. The lack of significant difference between the wildtype and Myd88^−/−^ neuronal type-1 IFN expression pattern at 48 h post-Aβ treatment can be explained by the existence of this wildtype biphasic response.

It is well accepted that a neuro-inflammatory environment, if not controlled, is deleterious to neuronal viability and disease progression. IL-1β, IL-6 and TNFα remain as hallmark pro-inflammatory cytokines that can trigger necrosis, apoptosis and excitotoxicity. We have shown that Myd88^−/−^ neuronal cultures, with reduced pro-inflammatory cytokine load, are protected against Aβ1-42 when compared to wildtype cells. These findings suggest that limiting the pro-inflammatory environment induced by amyloid, at least in a neuronal context, is beneficial in promoting neuro-protection. Neuro-inflammation is a complex but coordinated event that is tightly regulated by specific cellular processes but also the timing in which they occur. The timing of individual pro-inflammatory cytokine and chemokine release is crucial in recruitment of immune cells to the inflamed site and ensuring that this microenvironment facilitates an efficient immune response. Our data suggests that neurons, in response to amyloid, release an initial short-term interleukin pro-inflammatory component and potentiated TNFα component that correlates well with the time frame of Aβ1-42-induced neurotoxicity. Whilst IL-1β, IL-6 and TNFα pro-inflammatory cytokine secretions in neuro-inflammation share great interplay, the regulatory mechanisms governing their production differ [74-77]. Type-1 IFNs remain critical to the inflammatory response and can regulate the NALP3 inflammasome responsible for IL-1β production [78], previously implicated in AD pathology [10]. It remains unclear if alterations in type-1 IFN expression alone are sufficient to modulate the Aβ inflammasome or if other signalling pathways are involved, providing scope for further investigation.

Type-1 IFN production involves multiple signalling cascades. Although TLR activation is a primary source of type-1 IFNs [28], other pattern recognition receptors can also be responsible. Retinoic acid-inducible gene-1 (RIG-1), melanoma differentiation-associated protein-5 (MDA-5) and interferon gamma-inducible protein 16 (IFI-16) are all examples of TLR-independent activators of the type-1 IFN response [29]. Furthermore, complement can stimulate plasmacytoid dendritic cells to produce IFNα [79] and is a major component of the innate immune response. Despite this myriad of potential type-1 IFN secretion pathways, they all converge on the phosphorylation/activation of IRFs to initiate type-1 IFN gene transcription. Specifically, IRF7 is required for type-1 IFN production [61] and is an important regulator for many of the aforementioned innate immunity signalling cascades. Upon stimulation of human neuroblastoma M17 cell cultures with the TLR4 agonist LPS we found that IRF7 knockdown was sufficient to ablate the type-1 IFN response to the bacterial endotoxin. This data supports previous dogma that stimulation of TLR4 induces a type-1 IFN response through IRF7 activation [80,81]. Initially, this response is mediated by a TRIF-dependent interaction but subsequent type-1 IFN production remains Myd88 dependent [82]. LPS induces a well-characterised TLR4-mediated response and is useful as a pharmacological tool in this respect; however, Aβ as an endogenous pathogenic ligand interacts with numerous receptors associated with the innate immune system [19]. We identified that knockdown of IRF7 was sufficient to decrease type-1 IFN expression and downstream p-STAT-3 signalling in response to Aβ1-42. This similarity between the type-1 IFN release profile from cultures stimulated with either LPS or Aβ infers that TLR4 detection of amyloid contributes to the net type-1 IFN response. Under conditions of neuronal stress [83] or innate immune system priming [82], TLR4 signalling can induce a robust IRF7-dependent type-1IFN response. Our findings suggest that a TLR4-IRF7 driven type-1 IFN response is involved in the cellular response to soluble amyloid.

Alongside IRF7, IRF3 is also critical in producing a type-1 IFN response to pathogenic material [84]. TLR4 activation leads to the formation of the TBK1/IKKi complex and can phosphorylate both IRF3 [85] and IRF7 [86] leading to DNA binding and type-1 IFN transcription. Furthermore, activation of NFκB, downstream of TLR4, has been implicated in Aβ signalling and apoptotic neurons in human AD brains [87,88]. NFκB is capable of modulating both IRF3 and IRF7 activation via transcription and production of pro-inflammatory cytokines [89]. The precise contribution of TLR-mediated activation of IRF3 in the neuronal type-1 IFN response to amyloid and potential interplay with IRF7 has yet to be explored and warrants further investigation. It is likely that TLR4 binding is a significant component of Aβ-induced type-1 IFN production; however, the overall response is likely to be the summation of many activated sensors of the innate immune system.

Considering Myd88^−/−^ neurons secrete reduced type-1 IFN in response to Aβ and exhibit significant neuro-protection, we assessed viability of IRF7 knockdown M17 cultures. Similar to the Myd88^−/−^ cells, M17 IRF7 KD cells were protected against Aβ1-42-induced cytotoxicity. We therefore hypothesise that reducing neuronal type-1 IFN levels in response to Aβ will be beneficial for cellular survival. The current study uses the Aβ1-42 peptide as the sole ligand to trigger an innate immune response. As amyloid is a neurotoxic peptide, the neuronal cultures are under duress and can release DAMPs which are readily recognised by PRRs. The subsequent release of neuronal IL-1β, IL-6 and TNFα actively contributes to the neuro-inflammatory environment and drives excitotoxicity [90]. Despite amyloid-induced signalling occurring prior to that of DAMPs, it remains difficult to predict what proportion of the inflammatory response is induced purely by amyloid rather than cellular stress and debris. Delineating these pathways will be important in identifying the exact initial binding partners of Aβ and may reveal novel therapeutic targets.

The concept of inflammatory homeostasis controlled by neuronal reflex activity is well established with cholinergic activity being implicated in negatively regulating pro-inflammatory responses [91,92]. Indeed, genetic ablation of the α7 nicotinic acetylcholine (ACh) receptor (nAChR) in mice leads to loss of vagal nerve-induced suppression of TNFα [93] and α7 agonists can significantly inhibit the cytokine production induced by pathogenic TLR ligands [94]. Indeed, cholinergic neural activity activates the CREB/cfos transcriptional pathways leading to inhibition of NFκB in various innate and adaptive immune cells. TLR activation via various pathogens and Aβ is a major driver of NFκB activity, and thus these two signalling pathways compete against each other to govern the pro-inflammatory cytokine response to pathogenic insult and/or tissue damage [95]. In the context of AD, patients commonly display decreased cerebral and hippocampal α7 nAChR expression and cholinergic activity [96]. As mentioned previously, dysregulation of the cholinergic system can lead to a pro-inflammatory imbalance and this leads to declines in executive function and brain metabolism in mice [97]. MicroRNA-132 is a negative regulator of synaptic AChesterase [98] and has recently been identified as decreased in early stages of AD [99], suggesting that decreased cholinergic activity and hence elevated pro-inflammatory cytokine load is an early event contributing to disease progression. Findings from our study reveal a novel mechanism by which TLR signalling, in opposition to cholinergic activity, drives type-1 IFN production and pro-inflammatory cytokine secretion in response to Aβ. This finding highlights a potential mechanism by which neuro-inflammation is exacerbated in the absence of efficient cholinergic control in AD.

Whilst our findings support developing therapeutics which can modulate type-1 IFN levels in conditions of amyloidosis, agents targeting neuro-inflammation should be treated with caution. Type-1 IFNs are pleiotropic in nature and behave remarkably different depending on cell type. Indeed, type-1 IFNs are critical in viral immunity and beneficial inflammatory processes needed for clearance of amyloid. Targeting the TLR network therapeutically may prove a viable way of limiting type-1 IFN levels, but it is critical that the degree of modulation, the timing of therapeutic intervention and cell-type specificity be taken into account. In support of this, APP_swe_/PS1_ΔE9_ mice carrying a non-functional TLR4 mutant allele displayed decreased microglial activation and increased amyloid deposition and were more vulnerable to spatial learning memory deficits in the Morris water maze [26]. Furthermore, TLR4 is required for microglial phagocytosis of degenerating axons and efficient removal of cellular debris [83]. In addition, mice lacking TLR9 develop a spontaneous anxious phenotype due to lack of non-canonical NFκB activation, crucial in controlling anti-inflammatory cascades [100]. It is clear from these findings that complete removal of the TLR response remains deleterious due to suppression or lack of coordination in the immune response. Rather a fine balance is required where a neuro-inflammatory response is initiated, clearance of amyloid occurs and inflammation is resolved. In AD, where neuro-inflammation remains chronically activated, therapeutic intervention is required. Our findings identify that the neuronal type-1 IFN response to amyloid is deleterious to neuronal viability and that this response is TLR-Myd88-IRF7 dependent. This highlights a novel production pathway of the type-1 IFNs, a master regulator of innate immunity, in Aβ-induced neuro-inflammation. The neuronal origin of this finding highlights the importance in understanding the cell-specific responses to amyloid. This knowledge will be critical in identifying and targeting novel signalling pathways to limit both the neuro-inflammatory component and subsequent neuronal cell death of AD.

## Additional files

Additional file 1: Figure S1. Evaluation of IRF7 knockdown efficiency in human BE(2) M17 human neuroblastoma cells. M17 cells stably transfected with an IRF7 shRNA or corresponding negative control shRNA plasmid were analysed by Q-PCR to evaluate knockdown efficiencies. Data for IRF7 knockdown is expressed as fold change normalised to corresponding negative control transfected cells (nominal value of 1). Graphical data is displayed as mean ± SEM (*n* = 4). Additional file 2: Table S1. TLR 2/4 expression in wildtype and Myd88^−/−^ neurons. Un-treated, day 9 *in vitro*, wildtype and Myd88^−/−^ primary neuronal cultures were analysed by Q-PCR to determine TLR2 and TLR4 expression levels. Observed raw GAPDH, TLR2 and TLR4 Ct values are displayed and no reverse transcriptase (RT) and water controls remained undetermined (U.D.). Data is expressed as mean ± SEM (*n* = 4). Additional file 3: Figure S3. Full Western blot images of Aβ1-42-treated wildtype and Myd88^−/−^ neuronal cultures. Wildtype and Myd88^−/−^ primary cultured murine mixed cortical and hippocampal neuronal cultures were treated with 2.5 μM Aβ1-42 for 24 to 72 h. Western blotting, probing for p-STAT-3, STAT-3 and β-actin, was performed as described previously within the Materials and methods section. Bands of interest were selected from the full-sized Western blot image, as indicated by the red box, and cropped. This cropped image was then subjected to uniform image enhancement of contrast and brightness to yield the publication image seen in Figure 1C. Molecular weights were determined using the Precision Plus Protein™ WesternC Standard (Bio-Rad, 161-0376) that yields a colorimetric image only and has been removed from the chemiluminescent blot image. Additional file 4: Figure S2. Aβ42-1-treated neurons do not initiate a type-1 interferon response. Wildtype primary cultured murine mixed cortical and hippocampal neuronal cultures were treated with 2.5 μM Aβ42-1 (reverse sequence peptide) for 24 to 72 h. (A) IFNα and (B) IFNβ mRNA levels were then analysed by Q-PCR (*n* = 4). (B) Tyrosine 705 phosphorylation of STAT-3 was detected by Western blot in these same cultures. All graphical data is displayed as mean ± SEM with treatment time on the *x* axis. Additional file 5: Figure S4. The type-1 IFN response to Aβ1-42 is attenuated in an alternate clonal M17 IRF7 shRNA knockdown cell line. Human BE(2) M17 neuroblastoma cells, transfected with an IRF7 knockdown (KD) construct or negative control (NC) plasmid, were treated with 7.5 μM Aβ1-42 for 24 to 96 h. Q-PCR was performed to analyse (A) IFNα and (B) IFNβ mRNA levels post-treatment (**p* < 0.05, ***p* < 0.01, *n* = 3 to 4, unmatched two-way ANOVA, Bonferroni *post hoc* test). Data shown is from an alternative clonal cell line to that used in Figures 4 and 5. (C) Levels of IRF7 knockdown in these cells were also determined by Q-PCR where data is expressed as fold change normalised to corresponding negative control transfected cells (nominal value of 1, *n* = 3). Graphical data is displayed as mean ± SEM. Additional file 6: Figure S5. Full Western blot images of Aβ1-42-treated M17 NC and IRF7 KD cell cultures. Human BE(2) M17 neuroblastoma cells, transfected with an IRF7 knockdown (KD) construct or negative control (NC) plasmid, were treated with 7.5 μM Aβ1-42 for 24 to 96 h. Western blotting, probing for p-STAT-3, STAT-3 and β-actin, was performed as described previously within the Materials and methods section. Bands of interest were selected from the full-sized western blot image, as indicated by the red box, and cropped. This cropped image was then subjected to uniform image enhancement of contrast and brightness to yield the publication image seen in Figure 4E. Molecular weights were determined using the Precision Plus Protein™ WesternC Standard (Bio-Rad, 161-0376) that yields a colorimetric image only and has been removed from the chemiluminescent blot image. Additional file 7: Figure S6. Full Western blot images of LPS-treated M17 NC and IRF7 KD cell cultures. Human BE(2) M17 neuroblastoma cells, transfected with an IRF7 knockdown (KD) construct or negative control (NC) plasmid, were treated with 100 ng/ml LPS for 0.5 to 24 h. Western blotting, probing for p-STAT-3, STAT-3 and β-actin, was performed as described previously within the Materials and methods section. Bands of interest were selected from the full-sized Western blot image, as indicated by the red box, and cropped. This cropped image was then subjected to uniform image enhancement of contrast and brightness to yield the publication image seen in Figure 4H. Molecular weights were determined using the Precision Plus Protein™ WesternC Standard (Bio-Rad, 161-0376) that yields a colorimetric image only and has been removed from the chemiluminescent blot image.

## Abbreviations

Aβ: amyloid-beta
ACh: acetylcholine
AD: Alzheimer’s disease
ANOVA: analysis of variance
APP: amyloid precursor protein
BACE: beta-site amyloid precursor protein cleavage enzyme
BDNF: brain-derived neurotrophic factor
CNS: central nervous system
CREB: cAMP response element-binding protein
DAMP: damage-associated molecular pattern
DAPI: 4′,6-diamidino-2-phenylindole
DEPC: diethylpyrocarbonate
DNA: deoxyribonucleic acid
FBS: Foetal bovine serum
GFAP: glial fibrillary protein
IFI-16: interferon gamma-inducible protein-16
IFN: interferon
IKKi: inhibitor of NFκB kinase
IL: interleukin
IRF: interferon regulatory factor
JAK: Janus-associated kinase
KD: knockdown
LPS: lipopolysaccharide
MDA-5: melanoma differentiation-associated protein-5
Myd88: myeloid differentiation factor-88
MTS: 3-(4,5-dimethylthiazol-2-yl)-5-(3-carboxymethoxyphenyl)-2-(4-sulfophenyl)-2H-tetrazolium
nAChR: nicotinic acetylcholine receptor
NALP: nucleotide-binding oligomerisation receptor
NC: negative control
NeuN: hexaribonucleotide binding protein-3
NFκB: nuclear factor kappa-B
p-: phosphorylated
PBS: phosphate-buffered saline
PCR: polymerase chain reaction
PRR: pattern recognition receptor
PS: presenilin
PVDF: polyvinylidene fluoride
RIG-1: retinoic acid inducible gene-1
RNA: ribonucleic acid
SEM: standard error of the mean
STAT: signal transducer and activator of transcription
TBK-1: TANK-binding kinase-1
TBS-T: tris-buffered saline-Tween 20
TLR: toll-like receptor
TNF: tumour necrosis factor
TRIF: toll/interleukin-1-domain-containing adapter-inducing interferon-β
Tyk: tyrosine kinase
