# Supplementary figures and images for: Soluble amyloid triggers a myeloid differentiation factor 88 and interferon regulatory factor 7 dependent neuronal type-1 interferon response in vitro
Source: J Neuroinflammation. 2015 Apr 12;12:71. doi: 10.1186/s12974-015-0263-2 (PMC4407532; doi:10.1186/s12974-015-0263-2)

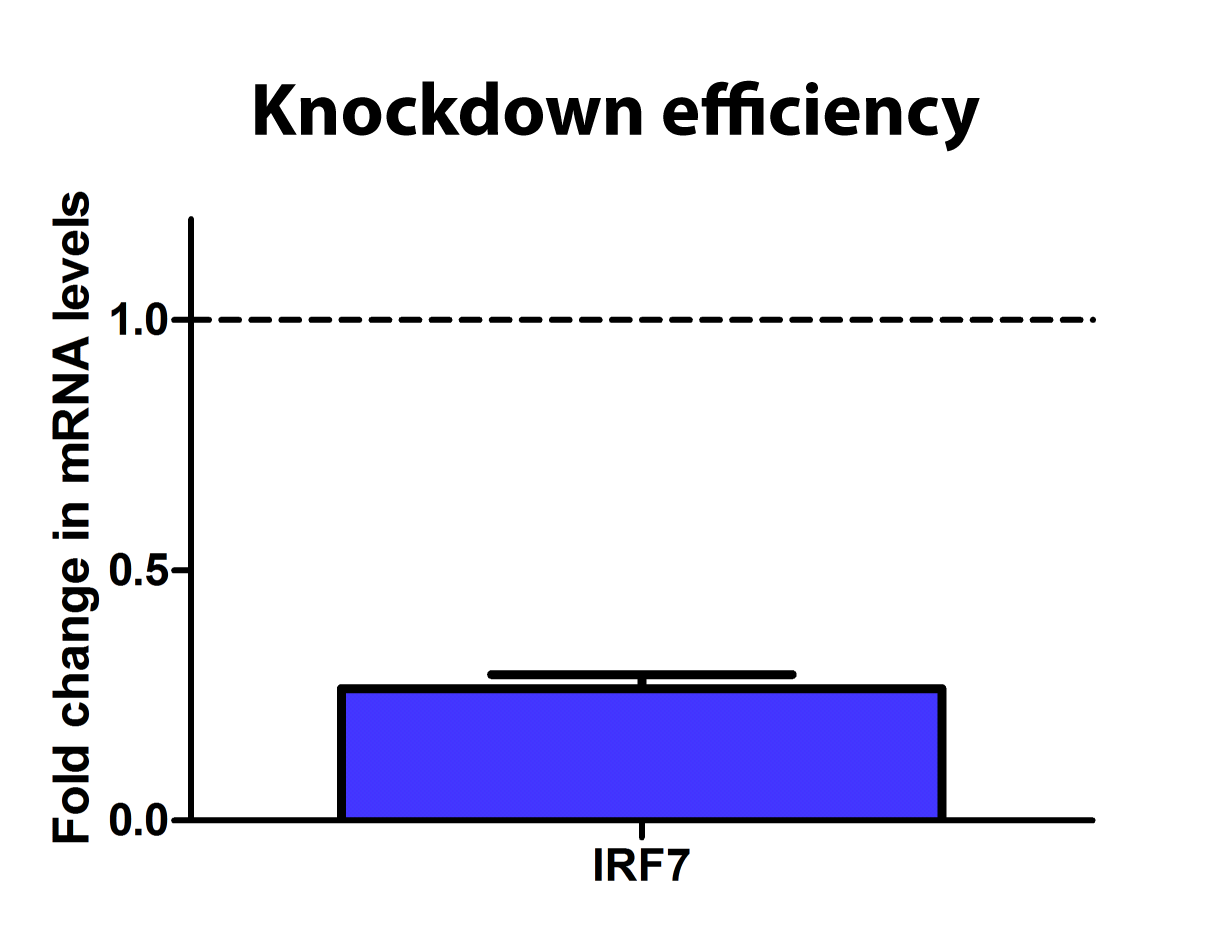

Supplement: Additional file 1: Figure S1. — Evaluation of IRF7 knockdown efficiency in human BE(2) M17 human neuroblastoma cells. M17 cells stably transfected with an IRF7 shRNA or corresponding negative control shRNA plasmid were analysed by Q-PCR to evaluate knockdown efficiencies. Data for IRF7 knockdown is expressed as fold change normalised to corresponding negative control transfected cells (nominal value of 1). Graphical data is displayed as mean ± SEM (n = 4). [file 12974_2015_263_MOESM1_ESM.tif]

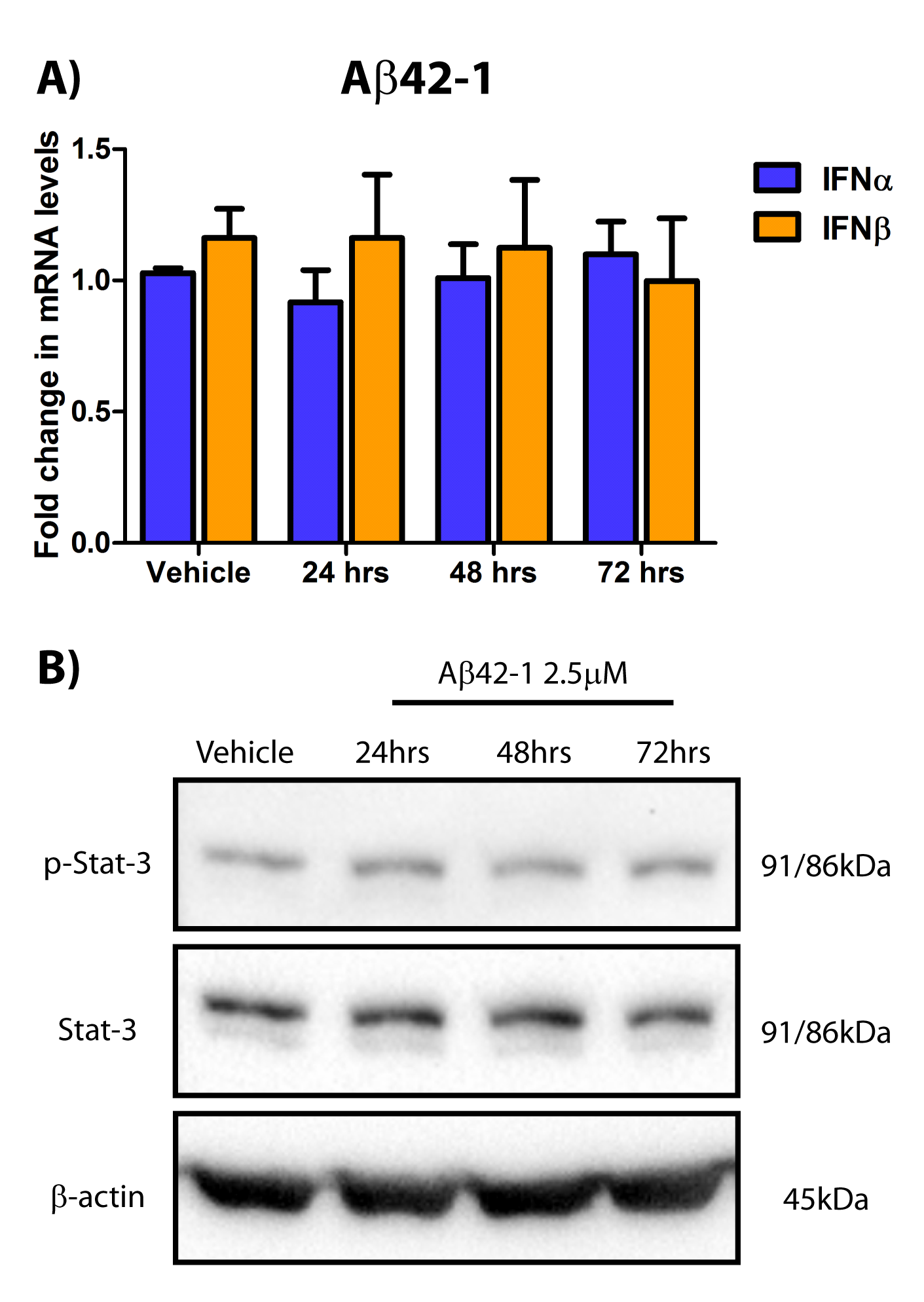

Supplement: Additional file 3: Figure S3. — Full Western blot images of Aβ1-42-treated wildtype and Myd88−/− neuronal cultures. Wildtype and Myd88−/− primary cultured murine mixed cortical and hippocampal neuronal cultures were treated with 2.5 μM Aβ1-42 for 24 to 72 h. Western blotting, probing for p-STAT-3, STAT-3 and β-actin, was performed as described previously within the Materials and methods section. Bands of interest were selected from the full-sized Western blot image, as indicated by the red box, and cropped. This cropped image was then subjected to uniform image enhancement of contrast and brightness to yield the publication image seen in Figure 1C. Molecular weights were determined using the Precision Plus Protein™ WesternC Standard (Bio-Rad, 161-0376) that yields a colorimetric image only and has been removed from the chemiluminescent blot image. [file 12974_2015_263_MOESM3_ESM.tif]

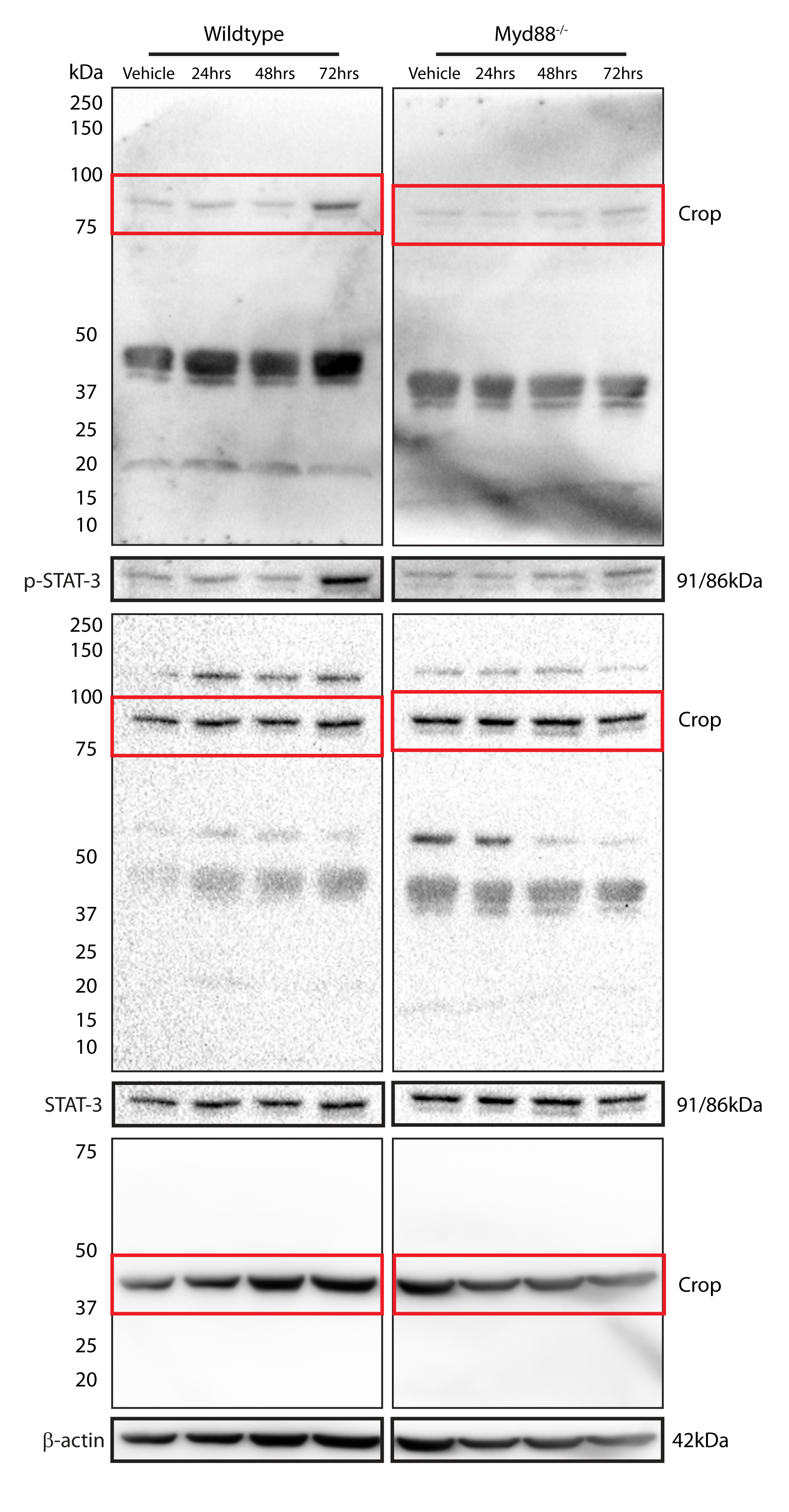

Supplement: Additional file 4: Figure S2. — Aβ42-1-treated neurons do not initiate a type-1 interferon response. Wildtype primary cultured murine mixed cortical and hippocampal neuronal cultures were treated with 2.5 μM Aβ42-1 (reverse sequence peptide) for 24 to 72 h. (A) IFNα and (B) IFNβ mRNA levels were then analysed by Q-PCR (n = 4). (B) Tyrosine 705 phosphorylation of STAT-3 was detected by Western blot in these same cultures. All graphical data is displayed as mean ± SEM with treatment time on the x axis. [file 12974_2015_263_MOESM4_ESM.tif]

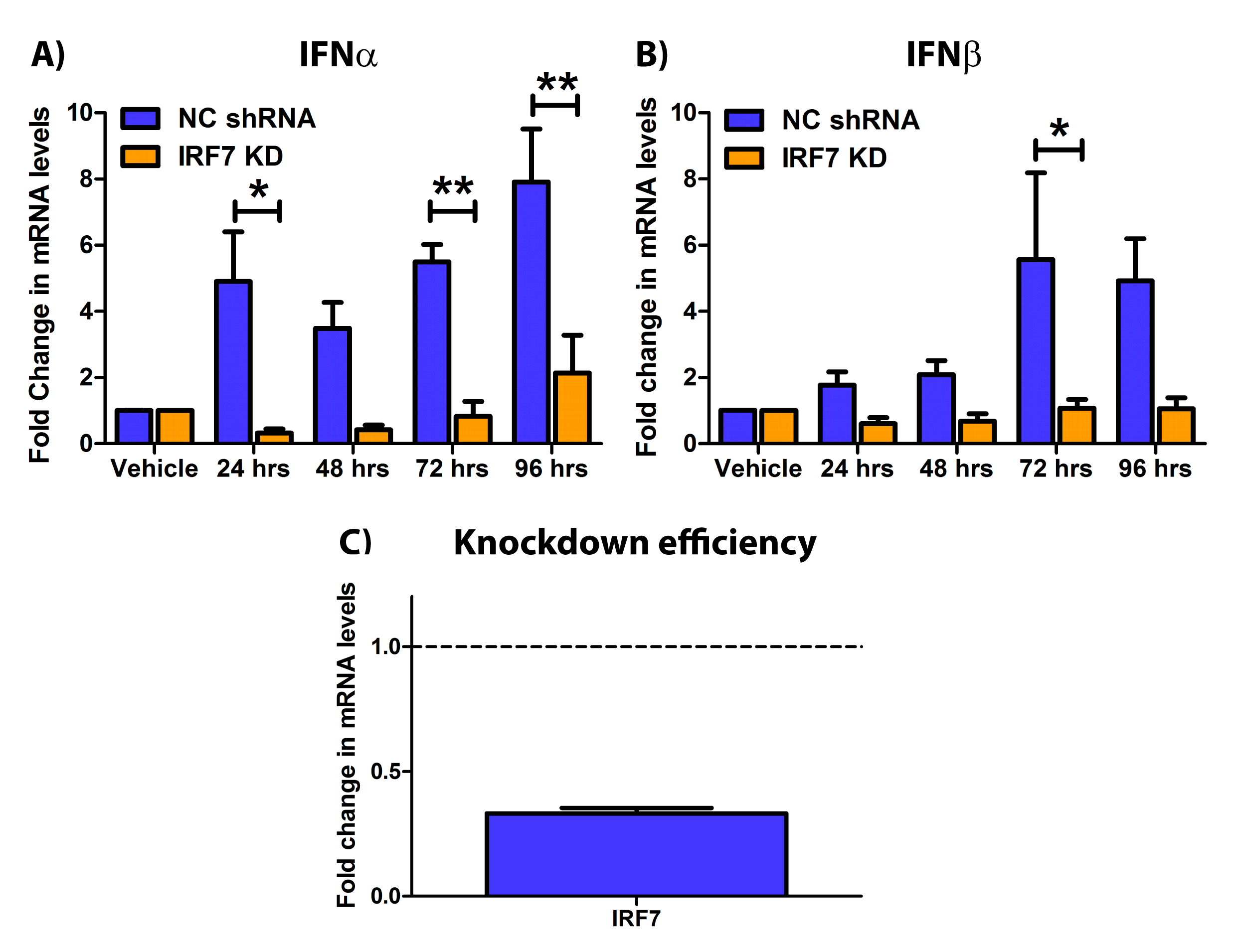

Supplement: Additional file 5: Figure S4. — The type-1 IFN response to Aβ1-42 is attenuated in an alternate clonal M17 IRF7 shRNA knockdown cell line. Human BE(2) M17 neuroblastoma cells, transfected with an IRF7 knockdown (KD) construct or negative control (NC) plasmid, were treated with 7.5 μM Aβ1-42 for 24 to 96 h. Q-PCR was performed to analyse (A) IFNα and (B) IFNβ mRNA levels post-treatment (*p < 0.05, **p < 0.01, n = 3 to 4, unmatched two-way ANOVA, Bonferroni post hoc test). Data shown is from an alternative clonal cell line to that used in Figures 4 and 5. (C) Levels of IRF7 knockdown in these cells were also determined by Q-PCR where data is expressed as fold change normalised to corresponding negative control transfected cells (nominal value of 1, n = 3). Graphical data is displayed as mean ± SEM. [file 12974_2015_263_MOESM5_ESM.tif]

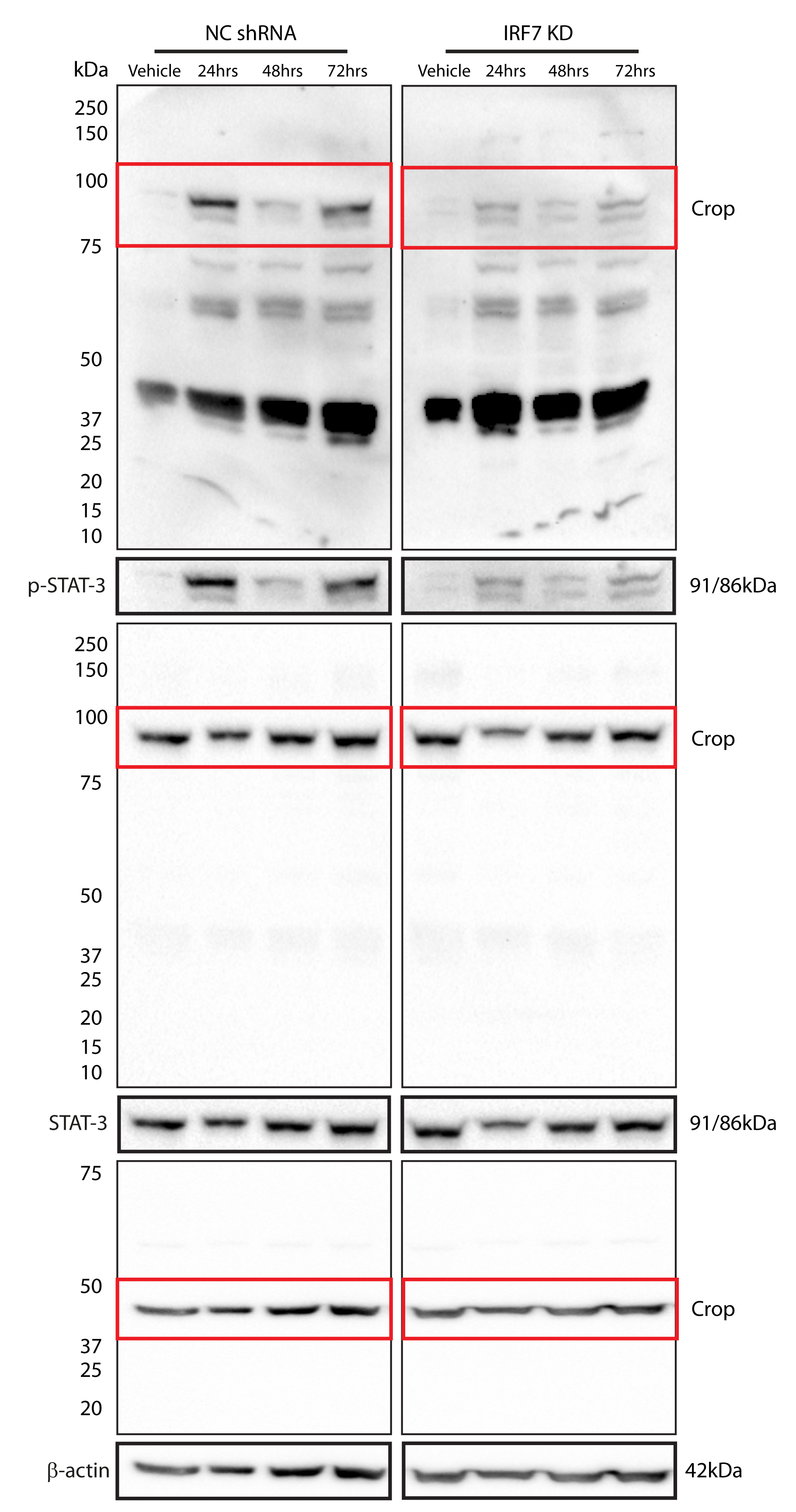

Supplement: Additional file 6: Figure S5. — Full Western blot images of Aβ1-42-treated M17 NC and IRF7 KD cell cultures. Human BE(2) M17 neuroblastoma cells, transfected with an IRF7 knockdown (KD) construct or negative control (NC) plasmid, were treated with 7.5 μM Aβ1-42 for 24 to 96 h. Western blotting, probing for p-STAT-3, STAT-3 and β-actin, was performed as described previously within the Materials and methods section. Bands of interest were selected from the full-sized western blot image, as indicated by the red box, and cropped. This cropped image was then subjected to uniform image enhancement of contrast and brightness to yield the publication image seen in Figure 4E. Molecular weights were determined using the Precision Plus Protein™ WesternC Standard (Bio-Rad, 161-0376) that yields a colorimetric image only and has been removed from the chemiluminescent blot image. [file 12974_2015_263_MOESM6_ESM.tif]

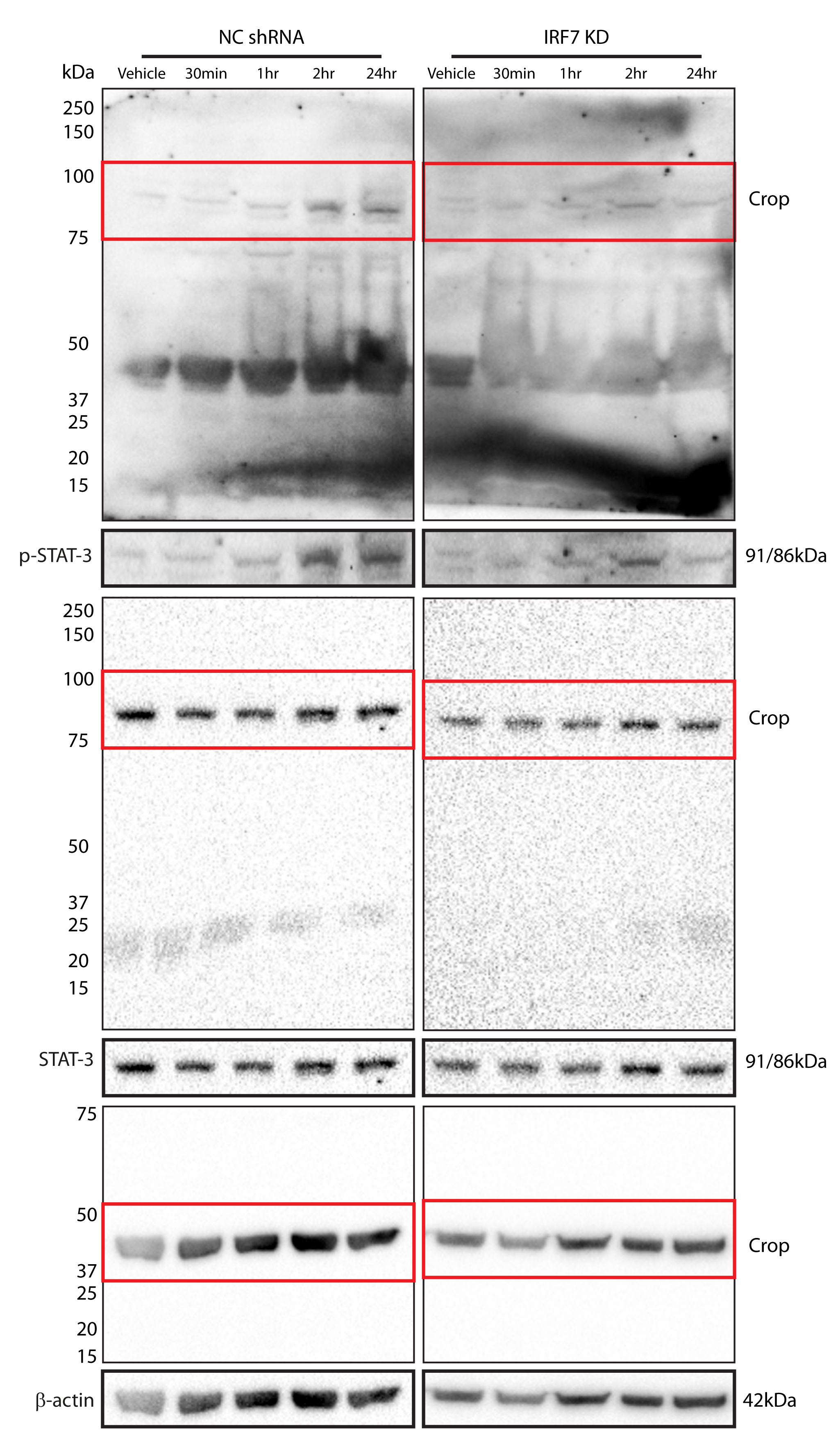

Supplement: Additional file 7: Figure S6. — Full Western blot images of LPS-treated M17 NC and IRF7 KD cell cultures. Human BE(2) M17 neuroblastoma cells, transfected with an IRF7 knockdown (KD) construct or negative control (NC) plasmid, were treated with 100 ng/ml LPS for 0.5 to 24 h. Western blotting, probing for p-STAT-3, STAT-3 and β-actin, was performed as described previously within the Materials and methods section. Bands of interest were selected from the full-sized Western blot image, as indicated by the red box, and cropped. This cropped image was then subjected to uniform image enhancement of contrast and brightness to yield the publication image seen in Figure 4H. Molecular weights were determined using the Precision Plus Protein™ WesternC Standard (Bio-Rad, 161-0376) that yields a colorimetric image only and has been removed from the chemiluminescent blot image. [file 12974_2015_263_MOESM7_ESM.tif]
